# Supplementary material for: Plasma Bile Acid Profiling and Modulation of Secreted Mucin 5AC in Cholangiocarcinoma
Source: Int J Mol Sci. 2023 Aug 14;24(16):12794. doi: 10.3390/ijms241612794 (PMC10454108; doi:10.3390/ijms241612794)
Supplement: Supplementary file 1 [file ijms-24-12794-s001.zip › ijms-2511294-supplementary.pdf]

Supplementary results

Clinical study

Supplemtary Table S1: laboratory data

	BBD	CCA
CA (umol/L)	0.28±1.12	0.13±0.28
GCA (umol/L)	1.01±2.55	14.77±31.80
TCA (umol/L)	0.38±1.42	9.66±21.0
CDCA (umol/L)	0.71±2.96	0.36±1.61
GCDCA (umol/L)	1.13±1.91	5.3±8.7
TCDCA (umol/L)	0.28±0.68	3.79±7.73
DCA (umol/L)	0.38±1.02	0.11±0.16
GDCA (umol/L)	0.22±0.43	0.2 ±0.3
TDCA (umol/L)	0.05±0.16	0.08±0.14
HDCA (umol/L)	0.02±0.01	0.01±0.008
UDCA (umol/L)	0.46±1.13	0.33±1.40
GUDCA (umol/L)	1.16±2.80	7.80±32.99
TUDCA (umol/L)	0.04±0.07	1.1±4.5
GLCA (umol/L)	0.01±0.02	0.02±0.03
TLCA (umol/L)	0.01±8.78	0.007±0.001
Total bilirubin (umol/L)	16.96±30.56	93.13±124.69
Total cholesterol (mmol/L)	4.41±1.17	4.57±1.59

Supplementray Table S2: logistic regression analysis. Full data.

_

. logistic Disease Gender Age tBil tCol P_TUDCA_uM P_TUDCA_uM_divchol

Disease	Odds Ratio	Std. Err.	z	P>|z|	[95% Conf.	Interval]
Gender	1.487824	.7509221	0.79	0.431	.5532741	4.000948
Age	1.061776	.0220192	2.89	0.004	1.019484	1.105822
tBil	1.018923	.0095633	2.00	0.046	1.000351	1.03784
tCol	.8824918	.2215503	-0.50	0.619	.5395302	1.443463
P_TUDCA_uM	6.88e+08	7.71e+09	1.82	0.069	.1977611	2.39e+18
P_TUDCA_uM_~l	1.90e-25	7.05e-24	-1.53	0.125	4.73e-57	7606687

. logistic Disease Gender Age tBil tCol P_GCDCA_uM P_GCDCA_uM_divchol

Disease	Odds Ratio	Std. Err.	z	P>|z|	[95% Conf.	Interval]
Gender	1.244926	.6168123	0.44	0.658	.4714182	3.287613
Age	1.064623	.0225743	2.95	0.003	1.021285	1.1098
tBil	1.017437	.0098222	1.79	0.073	.9983667	1.036871
tCol	.9367107	.2337183	-0.26	0.793	.5744107	1.527525
P_GCDCA_uM	2.0076	.8078164	1.73	0.083	.9123706	4.417565
P_GCDCA_uM_~l	.1435861	.1777318	-1.57	0.117	.0126908	1.624559

. logistic Disease Gender Age tBil tCol P_HDCA_uM P_HDCA_uM_divchol

Disease	Odds Ratio	Std. Err.	z	P>|z|	[95% Conf.	Interval]
Gender	1.292814	.6328153	0.52	0.600	.495318	3.374333
Age	1.064285	.0225344	2.94	0.003	1.021023	1.109381
tBil	1.02474	.0100979	2.48	0.013	1.005138	1.044723

tCol	1.412337	.4378057	1.11	0.265	.7692671	2.592982
P_HDCA_uM	1.29e-25	8.53e-24	-0.87	0.385	9.22e-82	1.81e+31
P_HDCA_uM_d~l	1.4e+109	3.4e+111	1.00	0.315	1.6e-104	.

. logistic Disease Gender Age tBil tCol P_GDCA_uM P_GDCA_uM_divchol

Disease	Odds Ratio	Std. Err.	z	P>|z|	[95% Conf.	Interval]
Gender	1.220472	.6128749	0.40	0.692	.456128	3.265646
Age	1.061228	.0219314	2.88	0.004	1.019102	1.105096
tBil	1.026187	.0101142	2.62	0.009	1.006554	1.046203
tCol	.9550095	.2296414	-0.19	0.848	.5961121	1.529986
P_GDCA_uM	36.42243	113.1937	1.16	0.247	.0824145	16096.59
P_GDCA_uM_d~l	3.90e-06	.0000381	-1.27	0.203	1.85e-14	819.6564

. logistic Disease Gender Age tBil tCol P_TLCA_uM P_TLCA_uM_divchol

Disease	Odds Ratio	Std. Err.	z	P>|z|	[95% Conf.	Interval]
Gender	1.195306	.5821995	0.37	0.714	.460133	3.105097
Age	1.059339	.0215451	2.83	0.005	1.017942	1.102419
tBil	1.026461	.0101967	2.63	0.009	1.00667	1.046642
tCol	.9478579	.4553283	-0.11	0.911	.3696974	2.430189
P_TLCA_uM	1	(omitted)				
P_TLCA_uM_d~l	3.6e-187	3.5e-184	-0.45	0.654	0	.

. logistic Disease Gender Age tBil tCol P_CDCA_uM P_CDCA_uM_divchol

Disease	Odds Ratio	Std. Err.	z	P>|z|	[95% Conf.	Interval]
Gender	1.321835	.6553857	0.56	0.574	.5001946	3.493139

```
         Age |   1.060104   .0218668     2.83   0.005       1.0181     1.10384
        tBil |   1.023627   .0097366     2.46   0.014     1.004721    1.042889
        tCol |   1.222397    .273406     0.90   0.369     .7885497    1.894939
   P_CDCA_uM |    .447403   .3441853    -1.05   0.296     .0990542    2.020807
P_CDCA_uM_d~l |   13.42105   34.21205     1.02   0.308     .0907735    1984.329


. logistic Disease Gender Age tBil tCol P_GLCA_uM P_GLCA_uM_divchol
------------------------------------------------------------------------------
     Disease | Odds Ratio   Std. Err.      z    P>|z|     [95% Conf. Interval]
-------------+----------------------------------------------------------------
      Gender |   1.185349   .5849455     0.34   0.730     .4506049    3.118148
         Age |   1.060405   .0217671     2.86   0.004     1.018589    1.103937
        tBil |   1.025922   .0101132     2.60   0.009     1.006291    1.045936
        tCol |   1.135514   .3194893     0.45   0.651     .6541802    1.971003
   P_GLCA_uM |   95486.68    5863234     0.19   0.852     5.16e-48    1.77e+57
P_GLCA_uM_d~l |   2.08e-08   4.43e-06    -0.08   0.934     1.1e-189    3.9e+173


. logistic Disease Gender Age tBil tCol P_DCA_uM P_DCA_uM_divchol
------------------------------------------------------------------------------
     Disease | Odds Ratio   Std. Err.      z    P>|z|     [95% Conf. Interval]
-------------+----------------------------------------------------------------
      Gender |   1.400463   .6940315     0.68   0.497     .5301992    3.699168
         Age |   1.060853   .0218617     2.87   0.004     1.018859    1.104578
        tBil |   1.021267   .0095627     2.25   0.025     1.002696    1.040183
        tCol |   1.245054   .3118186     0.88   0.381     .7620939    2.034081
    P_DCA_uM |   .0352341   .1184324    -1.00   0.320     .0000485    25.59205
P_DCA_uM_di~l |   4845.446   60680.71     0.68   0.498     1.06e-07    2.21e+14


. logistic Disease Gender Age tBil tCol P_GUDCA_uM P_GUDCA_uM_divchol
------------------------------------------------------------------------------
     Disease | Odds Ratio   Std. Err.      z    P>|z|     [95% Conf. Interval]
```

Gender	1.432727	.7109627	0.72	0.469	.5417154	3.78927
Age	1.062268	.0217696	2.95	0.003	1.020446	1.105804
tBil	1.02402	.0102868	2.36	0.018	1.004055	1.044381
tCol	1.043469	.2265019	0.20	0.845	.6818868	1.596786
P_GUDCA_uM	1.469297	.3651788	1.55	0.122	.9027177	2.391482
P_GUDCA_uM_~l	.3897057	.2815169	-1.30	0.192	.0945895	1.605575

. logistic Disease Gender Age tBil tCol P_TCA_uM P_TCA_uM_divchol

Disease	Odds Ratio	Std. Err.	z	P>|z|	[95% Conf.	Interval]
Gender	1.046574	.5661885	0.08	0.933	.3624735	3.021784
Age	1.0575	.0226896	2.61	0.009	1.013952	1.10292
tBil	1.056523	.0273975	2.12	0.034	1.004167	1.111609
tCol	.8137209	.2030032	-0.83	0.409	.4990243	1.326873
P_TCA_uM	8.736107	7.687534	2.46	0.014	1.556983	49.01759
P_TCA_uM_di~l	.0008951	.0023803	-2.64	0.008	4.88e-06	.1642278

. logistic Disease Gender Age tBil tCol P_GCA_uM P_GCA_uM_divchol

Disease	Odds Ratio	Std. Err.	z	P>|z|	[95% Conf.	Interval]
Gender	1.160118	.6141211	0.28	0.779	.411064	3.274123
Age	1.062807	.0232909	2.78	0.005	1.018124	1.109451
tBil	1.034883	.0167565	2.12	0.034	1.002556	1.068252
tCol	.7961627	.1975671	-0.92	0.358	.4895272	1.294872
P_GCA_uM	2.557007	.8736678	2.75	0.006	1.308871	4.995362
P_GCA_uM_di~l	.0493168	.0515023	-2.88	0.004	.006369	.3818727

. logistic Disease Gender Age tBil tCol P_TCDCA_uM P_TCDCA_uM_divchol

Disease	Odds Ratio	Std. Err.	z	P>|z|	[95% Conf.	Interval]
Gender	1.067481	.5697518	0.12	0.903	.3750115	3.038617
Age	1.065074	.0233519	2.88	0.004	1.020274	1.11184
tBil	1.044899	.0208679	2.20	0.028	1.004789	1.08661
tCol	.7926064	.1941659	-0.95	0.343	.4903855	1.281084
P_TCDCA_uM	42.28531	53.4915	2.96	0.003	3.543299	504.6279
P_TCDCA_uM_~l	1.76e-06	8.11e-06	-2.88	0.004	2.15e-10	.0144931

. logistic Disease Gender Age tBil tCol P_TDCA_uM P_TDCA_uM_divchol

Disease	Odds Ratio	Std. Err.	z	P>|z|	[95% Conf.	Interval]
Gender	1.114594	.5671763	0.21	0.831	.4111226	3.021775
Age	1.061421	.0221364	2.86	0.004	1.018909	1.105706
tBil	1.023659	.0097193	2.46	0.014	1.004786	1.042887
tCol	.9195044	.2151419	-0.36	0.720	.5812904	1.454502
P_TDCA_uM	1.66e+10	2.23e+11	1.75	0.080	.0611057	4.52e+21
P_TDCA_uM_d~l	2.32e-35	1.15e-33	-1.61	0.107	1.72e-77	3.12e+07

. logistic Disease Gender Age tBil tCol P_UDCA_uM P_UDCA_uM_divchol

Disease	Odds Ratio	Std. Err.	z	P>|z|	[95% Conf.	Interval]
Gender	1.321228	.6478568	0.57	0.570	.5053538	3.454297
Age	1.062106	.0217323	2.94	0.003	1.020355	1.105566
tBil	1.025708	.0100661	2.59	0.010	1.006167	1.045628
tCol	1.03132	.2274149	0.14	0.889	.6694164	1.588879
P_UDCA_uM	1.869566	1.015608	1.15	0.249	.6446749	5.421767
P_UDCA_uM_d~l	.0690215	.1739066	-1.06	0.289	.0004946	9.631031

. logistic Disease Gender Age tBil tCol P_CA_uM P_CA_uM_divchol

Disease	Odds Ratio	Std. Err.	z	P>|z|	[95% Conf.	Interval]
Gender	1.256455	.6146545	0.47	0.641	.4816609	3.277574
Age	1.063076	.0218999	2.97	0.003	1.021008	1.106878
tBil	1.02606	.0100892	2.62	0.009	1.006475	1.046026
tCol	1.051062	.2430258	0.22	0.829	.6680582	1.653646
P_CA_uM	2.31976	4.650016	0.42	0.675	.0456235	117.9499
P_CA_uM_div~l	.0096176	.0723045	-0.62	0.537	3.84e-09	24117.52

. logistic Disease Gender Age tBil tCol P_PriNon_uM P_PriNon_uM_divchol
PriNon = Primary unconjugated (sum umol/L)

Disease	Odds Ratio	Std. Err.	z	P>|z|	[95% Conf.	Interval]
Gender	1.317002	.6500625	0.56	0.577	.5005409	3.46524
Age	1.06008	.0217952	2.84	0.005	1.018211	1.10367
tBil	1.023923	.0097886	2.47	0.013	1.004916	1.043289
tCol	1.204558	.2701185	0.83	0.407	.776155	1.869421
P_PriNon_uM	.6284793	.3187501	-0.92	0.360	.2325848	1.698246
P_PriNon_uM~l	4.361172	7.353024	0.87	0.382	.1601255	118.7808

. logistic Disease Gender Age tBil tCol P_PriCon_uM P_PriCon_uM_divchol
PriCon = Primary conjugated (sum umol/L)

Disease	Odds Ratio	Std. Err.	z	P>|z|	[95% Conf.	Interval]
Gender	1.141838	.6037325	0.25	0.802	.4050813	3.218597
Age	1.065158	.023283	2.89	0.004	1.020487	1.111783
tBil	1.033474	.0161991	2.10	0.036	1.002207	1.065716

tCol	.732551	.1899713	-1.20	0.230	.4406534	1.217807
P_PriCon_uM	1.498849	.2060928	2.94	0.003	1.144767	1.96245
P_PriCon_uM~l	.2667806	.1186529	-2.97	0.003	.1115771	.6378716

. logistic Disease Gender Age tBil tCol P_SecNon_uM P_SecNon_uM_divchol
SecNon = Secondary unconjugated (sum umol/L)

Disease	Odds Ratio	Std. Err.	z	P>|z|	[95% Conf.	Interval]
Gender	1.287255	.628073	0.52	0.605	.4947081	3.349499
Age	1.06168	.0216697	2.93	0.003	1.020046	1.105012
tBil	1.025293	.0100194	2.56	0.011	1.005842	1.04512
tCol	1.029145	.231119	0.13	0.898	.6627029	1.598211
P_SecNon_uM	1.540673	.6893661	0.97	0.334	.6409805	3.703191
P_SecNon_uM~l	.1279891	.2533277	-1.04	0.299	.0026448	6.193678

. logistic Disease Gender Age tBil tCol P_SecCon_uM P_SecCon_uM_divchol
SecCon = Secondary conjugated (sum umol/L)

Disease	Odds Ratio	Std. Err.	z	P>|z|	[95% Conf.	Interval]
Gender	1.399684	.6916676	0.68	0.496	.531374	3.686885
Age	1.061957	.0217823	2.93	0.003	1.020111	1.105519
tBil	1.023431	.0101835	2.33	0.020	1.003665	1.043586
tCol	1.011061	.2249655	0.05	0.961	.6537043	1.563772
P_SecCon_uM	1.497463	.4223455	1.43	0.152	.8615539	2.602733
P_SecCon_uM~l	.348029	.3186971	-1.15	0.249	.05783	2.094487

. logistic Disease Gender Age tBil tCol P_PriTOSec P_PriTOSec_divchol
PriTOSec = Primary/Secondary (ratio)

```
      Disease | Odds Ratio   Std. Err.      z    P>|z|     [95% Conf. Interval]
--------------+----------------------------------------------------------------
       Gender |   1.128632   .6033478     0.23   0.821     .3958352    3.218033
          Age |    1.05706   .0228096     2.57   0.010     1.013286    1.102725
         tBil |   1.044486   .0164688     2.76   0.006     1.012701    1.077268
         tCol |   .8864384   .2114377    -0.51   0.613     .5554126    1.414756
    P_PriTOSec |   1.136914   .0543545     2.68   0.007      1.03522    1.248598
P_PriTOSec_~l |   .7200703   .0824055    -2.87   0.004     .5753904    .9011296


. logistic Disease Gender Age tBil tCol P_ConTONon P_ConTONon_divchol
ConTONon = Conjugated/Unconjugated (ratio)
-------------------------------------------------------------------------------
      Disease | Odds Ratio   Std. Err.      z    P>|z|     [95% Conf. Interval]
--------------+----------------------------------------------------------------
       Gender |   1.304357   .6755412     0.51   0.608     .4726606     3.59951
          Age |   1.057648   .0220768     2.69   0.007     1.015251    1.101815
         tBil |   1.012371   .0091829     1.36   0.175     .9945323    1.030531
         tCol |   .8391407   .1997848    -0.74   0.461     .5262336    1.338108
   P_ConTONon |    1.06443   .0332679     2.00   0.046     1.001183    1.131673
P_ConTONon_~l |   .8321488   .0755121    -2.02   0.043     .6965623    .9941272


. logistic Disease Gender Age tBil tCol P_PriNonTOSecNon P_PriNonTOSecNon_divchol
PriNonTOSecNon = Primary unconjugated/Secondary unconjugated (ratio)
-------------------------------------------------------------------------------
      Disease | Odds Ratio   Std. Err.      z    P>|z|     [95% Conf. Interval]
--------------+----------------------------------------------------------------
       Gender |   1.151389   .5740989     0.28   0.777      .433312    3.059449
          Age |   1.065069   .0223586     3.00   0.003     1.022136    1.109805
         tBil |   1.033495   .0117503     2.90   0.004      1.01072    1.056784
         tCol |   .9735693   .2227342    -0.12   0.907     .6217676    1.524424
P_PriNonTOS~n |   1.366737   .2482283     1.72   0.085     .9573902    1.951108
P_PriNonTOS~l |   .3441959   .1875053    -1.96   0.050      .118332    1.001173
```

```
. logistic Disease Gender Age tBil tCol P_PriConTOSecCon P_PriConTOSecCon_divchol
```

PriConTOSecCon = Primary conjugated/Secondary conjugated (ratio)

Disease	Odds Ratio	Std. Err.	z	P>|z|	[95% Conf.	Interval]
Gender	1.123182	.6007097	0.22	0.828	.3937342	3.204033
Age	1.056862	.0227576	2.57	0.010	1.013186	1.102421
tBil	1.045805	.0170301	2.75	0.006	1.012953	1.079722
tCol	.8880469	.2125631	-0.50	0.620	.5555102	1.419645
P_PriConTOS~n	1.115581	.0448277	2.72	0.006	1.031091	1.206994
P_PriConTOS~l	.7571491	.0735752	-2.86	0.004	.625845	.9160012

```
. logistic Disease Gender Age tBil tCol P_PriConTOPriNon P_PriConTOPriNon_divchol
```

PriConTOPriNon = Primary conjugated/Primary unconjugated (ratio)

Disease	Odds Ratio	Std. Err.	z	P>|z|	[95% Conf.	Interval]
Gender	1.292332	.6683468	0.50	0.620	.4689901	3.561101
Age	1.053073	.0219449	2.48	0.013	1.010928	1.096975
tBil	1.010151	.0091132	1.12	0.263	.9924464	1.028171
tCol	.8163938	.2007756	-0.82	0.409	.5041547	1.322013
P_PriConTOP~n	1.029373	.0166012	1.80	0.073	.9973438	1.06243
P_PriConTOP~l	.9188195	.0430002	-1.81	0.070	.8382904	1.007085

```
. logistic Disease Gender Age tBil tCol P_SecConTOSecNon P_SecConTOSecNon_divchol
```

SecConTOSecNon = Secondary conjugated/Secondary unconjugated (ratio)

Disease	Odds Ratio	Std. Err.	z	P>|z|	[95% Conf.	Interval]
Gender	1.160563	.5737172	0.30	0.763	.4404358	3.058124

Age	1.063218	.0222908	2.92	0.003	1.020414	1.107817
tBil	1.018404	.0098882	1.88	0.060	.9992066	1.03797
tCol	1.107179	.2375496	0.47	0.635	.7270918	1.685957
P_SecConTOS~n	1.065766	.0484206	1.40	0.161	.9749661	1.165022
P_SecConTOS~l	.8807741	.1819282	-0.61	0.539	.5875498	1.320336

_

In vitro study

Anti-MUC5AC antibody testing

As shown in Figure **1S**, panel A, left side, antibody against MUC5AC recognized a major isoform of 270 kDa in HuH28 lysates, also detected in human primary cholangiocytes, although less represented. Moreover, faint isoforms of about 470, 450, 230 and 100 kDa were detected in both cell lines. Additional faint bands of about 180 and 150 kDa were observed only in HuH28 lysate, while faint bands of 200 and 75 kDa were detected only in primary cholangiocytes lysate. All these isoforms could represent possible proteolytic cleavages and or products of multiple-stage glycosylation. The reactivity of anti-Rabbit-HRP secondary antibody alone to the cellular lysates was tested confirming antibody specificity to the mentioned bands (figure 4 panel A, right side). The only bands detected by secondary antibody alone included a very faint band at about 280 kDa in HuH28 and a band of about 50 kDa present in both cell-type lysates and therefore considered unspecific. Extracts from A549 cells derived from airway epithelium and used here as positive control for our anti-MUC5AC antibody, in addition to a predominant large range of isoforms above 500 kDa we also observed the 470, 450, 270, 230 and 100 kDa bands detected in HuH28 and cholangiocytes. Anti-MUC5AC was also tested on collected media from HuH28 and primary cholangiocytes cultures. As shown in Figure **1S**, panel B, left side, antibody against MUC5AC labelled a 270 kDa secreted isoform in both cell lines (black arrow), but less expressed in primary cholangiocytes. In addition, bands of about 50 and 100 kDa were detected. Incubation of the same samples with secondary antibodies only showed the disappearance of the 270 kDa band, indicating it is specific, while the 50 and 100 kDa bands were labelled, suggesting they are unspecific (panel B, right side).

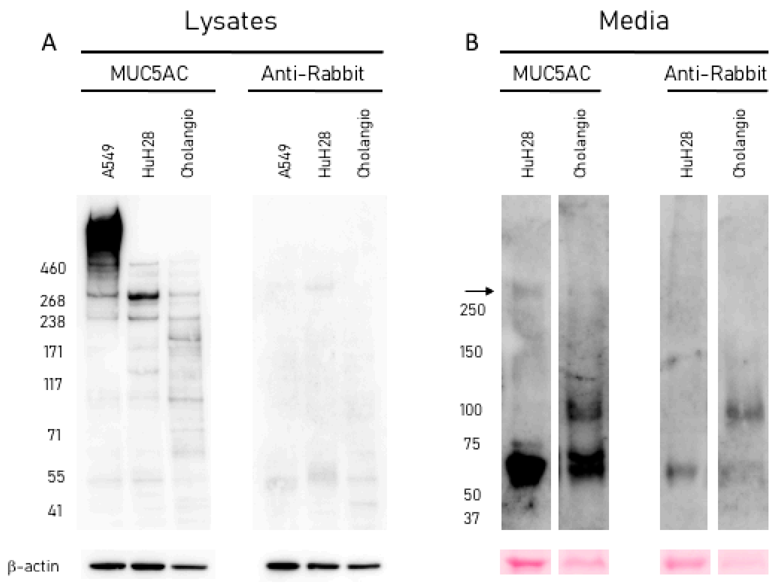

Figure S1. MUC5AC expression profile in HuH28 and cholangiocytes lysates and cell culture media. Panel A: incubation of lysates with anti-MUC5AC antibody (left side), incubation with secondary antibody only (right side). 10 µg of HuH28 and cholangiocytes lysates were loaded per lane, 1µg for A549 extracts. Panel B: incubation of collected media with anti-MUC5AC antibody (left side), incubation with secondary antibody only (right side). Lower panels: incubation with anti-β-actin, as a loading control for lysates and incubation with Ponceau solution for the media.

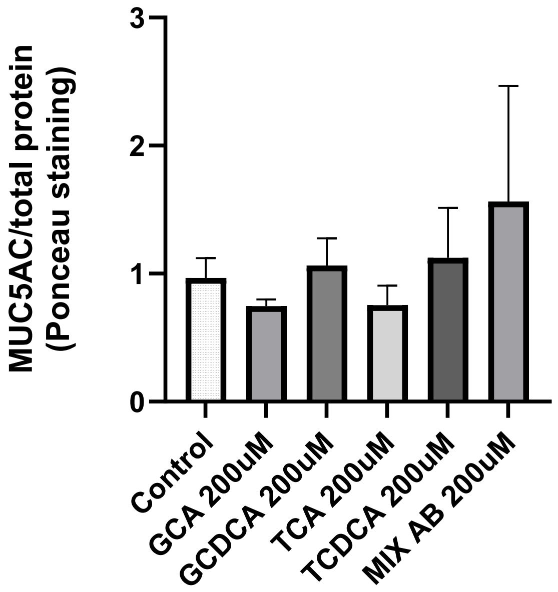

Figure S2. Quantification of MUC5AC expression in HuH28. Western blot signals and Ponceau staining were acquired by Image J software. Each 270 kDa band detected by the anti-MUC5AC antibody (from figure 4, left panel) was normalized for the respective Ponceau staining. Values obtained from the biliary acids treated samples were statistically compared to the untreated sample using One-way ANOVA test (GraphPad Prism 10 software), showing no significant differences. The graph plots the mean and standard deviation of three independent experiments.

.
